# Visual Display of 5p-arm and 3p-arm miRNA Expression with a Mobile Application

**DOI:** 10.1155/2017/6037168

**Published:** 2017-02-08

**Authors:** Chao-Yu Pan, Wei-Ting Kuo, Chien-Yuan Chiu, Wen-chang Lin

**Affiliations:** ^1^Institute of Biomedical Informatics, National Yang-Ming University, Taipei 112, Taiwan; ^2^Institute of Biomedical Sciences, Academia Sinica, Taipei 115, Taiwan; ^3^Institute of Biotechnology in Medicine, National Yang-Ming University, Taipei 112, Taiwan

## Abstract

MicroRNAs (miRNAs) play important roles in human cancers. In previous studies, we have demonstrated that both 5p-arm and 3p-arm of mature miRNAs could be expressed from the same precursor and we further interrogated the 5p-arm and 3p-arm miRNA expression with a comprehensive arm feature annotation list. To assist biologists to visualize the differential 5p-arm and 3p-arm miRNA expression patterns, we utilized a user-friendly mobile App to display. The Cancer Genome Atlas (TCGA) miRNA-Seq expression information. We have collected over 4,500 miRNA-Seq datasets from 15 TCGA cancer types and further processed them with the 5p-arm and 3p-arm annotation analysis pipeline. In order to be displayed with the RNA-Seq Viewer App, annotated 5p-arm and 3p-arm miRNA expression information and miRNA gene loci information were converted into SQLite tables. In this distinct application, for any given miRNA gene, 5p-arm miRNA is illustrated on the top of chromosome ideogram and 3p-arm miRNA is illustrated on the bottom of chromosome ideogram. Users can then easily interrogate the differentially 5p-arm/3p-arm expressed miRNAs with their mobile devices. This study demonstrates the feasibility and utility of RNA-Seq Viewer App in addition to mRNA-Seq data visualization.

## 1. Introduction

MicroRNAs (miRNAs) have been extensively studied and demonstrated to be potential biomarkers for human cancers [1–5]. They are short RNA molecules (around 22 nucleotides in length) and derived from endogenous nonprotein-coding transcripts [6, 7]. In their biogenesis processes, transcribed miRNAs precursors are subsequently cleaved by Drosha and Dicer enzymes to generate a mature miRNA duplex [8–11], and one strand of the miRNA duplex is preferentially selected to be the mature miRNA molecule and to form the ultimate RNA-induced silencing complex with Argonaute proteins [12, 13]. However, more and more next-generation sequencing (NGS) data suggests that both 5p-arm and 3p-arm mature miRNA on the duplex could be expressed and utilized [14–18]. It is essential to investigate the expression between 5p-arm and 3p-arm miRNA more thoughtfully. Thus, our laboratory has previously reported a comprehensive arm feature annotation pipeline for assisting the investigation of intrinsic properties of 5p-arm and 3p-arm miRNAs [19]. Interestingly, dominant 5p-arm or 3p-arm mature miRNA expression pattern is often noted [12, 20–22]. It would be beneficial to interrogate the expression of all 5p-arm and 3p-arm miRNAs in a systematic manner. Here, we tried to utilize a mobile App to visually display the 5p-arm and 3p-arm miRNA expression information in a user-friendly environment.

In recent years, mobile computing is a new trend in the Information Technology sector and it is worth noting that tablets and smartphones are widely popularized and disseminated on the world, replacing the traditional personal computers. Besides their portability and convenience, these devices also utilize the multitouch technology as the core user interface and gradually transform the user experience and machine interface design. The significant growth of mobile devices also greatly stimulates the software development of mobile applications (Apps) designed for mobile devices. Regrettably, very few mobile Apps were created to visualize human genome information as well as the NGS sequence data information besides eHealth Apps for introduction and education purposes. With the marked advance of computation capability and graphics processing units, in addition to screen size and resolution, tablets will be an excellent and suitable instrument for clinical information exchange and education purpose [23–25]. Previously, we have demonstrated the feasibility of visualization of RNA-Seq gene expression information on the mobile devices with a mobile App, RNA-Seq Viewer. In this report, we have now processed around 5000 TCGA (The Cancer Genome Atlas) miRNA-Seq expression data with 5p-arm and 3p-arm annotations and convert them to be displayed on the iPad platform with our RNA-Seq Viewer App. The iOS platform was chosen due to the dominant user usage share in the US and worldwide for tablets. In addition, our main purpose is to demonstrate the feasibility of NGS data interrogation on mobile devices. There are many hardware issues in the Android devices in terms of compatibility and performance because of diverse device manufactures and OS versions. Apple's iOS system seems to be more uniform and with better graphical capability, which is essential for large NGS data. Certainly, with the advance of Android OS and hardware, future development on RNA_Seq Viewer will be for both platforms.

## 2. Material and Methods

### 2.1. RNA-Seq Viewer

The development and implement of RNA_Seq Viewer was done using the Apple development software, Xcode 5.02. The programming language used was Object-C and database used was SQLite. The RNA_Seq Viewer App can be obtained freely through Apple iTune App Store (https://itunes.apple.com/us/app/rna-seq-viewer/id898456094?mt=8).

Four tables were designed to store the gene information, clinical data, and expression values. The description of each column in each table is listed below:

gene: geneName; name; chrom; strand; txStart; txEnd; scrX; scrY
normal: geneName; nr; nrlog
tumor: bcld; geneName; tr; trlog
sample: barcode; bcld; age; gender; pm; pn; pt; flag

Additional user information about the RNA-Seq Viewer can be accessed at http://tdl.ibms.sinica.edu.tw/NGS_viewer_1/NGS_Viewer/General_Introduction.html.

### 2.2. TCGA miRNA-Seq Data

We obtained the level three miRNA-Seq data as well as clinical information from TCGA website (https://gdc-portal.nci.nih.gov). The 15 TCGA cancer type datasets retrieved included bladder urothelial carcinoma (BLCA), breast invasive carcinoma (BRCA), colon adenocarcinoma (COAD), head and neck squamous cell carcinoma (HNSC), kidney chromophobe carcinoma (KICH), kidney renal clear cell carcinoma (KIRC), kidney renal papillary cell carcinoma (KIRP), liver hepatocellular carcinoma (LIHC), lung adenocarcinoma (LUAD), lung squamous cell carcinoma (LUSC), prostate adenocarcinoma (PRAD), rectum adenocarcinoma (READ), stomach adenocarcinoma (STAD), thyroid carcinoma (THCA), and uterine corpus endometrial carcinoma (UCEC). In summary, we obtained 4,625 tumor samples and 600 normal (adjacent tumor) samples. All datasets were processed and calculated for total read counts and RPM values (reads per million). The 5p-arm and 3p-arm were annotated and assigned using our refined arm feature list based on the mapping positions. During the processing, we excluded few TCGA sample libraries with less than one million reads. Therefore, the numbers of samples available for final presentation and download is slightly less than the numbers of samples retrieved from TCGA. Since the matching normal tissues were not always available in TCGA, we used the average expression values from all available normal tissues for that particular cancer type to represent the background expression levels and stored them in the “normal” table. Therefore, each cancer type will use only one normal expression value in this App.

## 3. Results and Discussion

### 3.1. TCGA miRNA-Seq Datasets

TCGA is a well-known large-scale cancer research program utilizing the high-throughput NGS sequencing platforms to accelerate the discovery of the molecular alterations and biomarkers of human cancers [26, 27]. We have now retrieved miRNA-Seq miRNA expression information from thousands of cancer patients in various cancer types collected in TCGA project. As described earlier, both 5p-arm and 3p-arm of mature miRNA duplex could be processed during biogenesis [16]. However, earlier literature reports and database annotations seldom use clear 5p-arm and 3p-arm annotations. This could result in ambiguous miRNA data interpretations, especially without clear indication of the arm features. Therefore, we have refined the miRNA annotation to clearly annotate the 5p-arm and 3p-arm feature on all human mature miRNAs obtained from miRBase database [28]. Our annotation list has improved the arm feature assignment rate from 50% to 99.9% [19].

We then used this refined annotation list to process TCGA miRNA-Seq datasets. In total, we have obtained 15 cancer types with 4,625 cancer tissue samples and 600 normal (adjacent tumor) tissues. We subsequently generated complete 5p-arm and 3p-arm miRNA expression profiles [29]. Our data could achieve more comprehensive analysis on dys-regulated miRNAs in cancer samples. This is especially important for NGS miRNA analysis, since more miRNA reads from both 5p-arm and 3p-arm could be observed with the increasing depth of sequencing. Since TCGA pipeline did not resolve the miRNA arm annotation and opposite arm miRNAs in the released level 3 information, we could improve miRNA expression information and provided extended understanding on opposite arm miRNA expression by utilizing our comprehensive miRNA arm feature annotations [29]. In this report, we would like to further improve data visualization on 5p-arm and 3p-arm miRNAs by using a mobile App.

### 3.2. Features of RNA-Seq Viewer App

**Figure 1:**
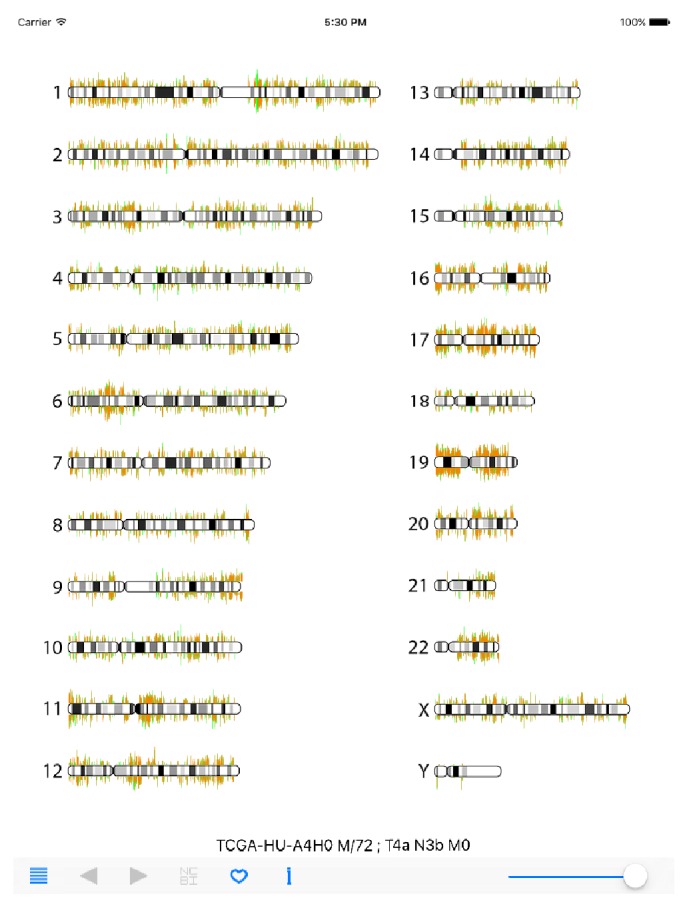
RNA-Seq Viewer display (overall chromosome view). Human gene expression information is displayed with the RNA-Seq Viewer App. Sample information and navigation panel are located at the bottom section. Tumor tissue expression is illustrated with light-red color lines and normal tissue expression background is illustrated with light-green color lines.

Our RNA_Seq Viewer is a mobile application aimed to provide a new user interface in examining NGS data intuitively for biologist and medical researchers. It displays expression information systematically on the chromosome level. An illustration of 22 autosomes and two sex chromosomes is displayed when the App is initiated (Figure 1). Users could easily interrogate differentially expressed genes by examining particular regions from the overall chromosome display view on their preferred mobile devices.

**Figure 2:**
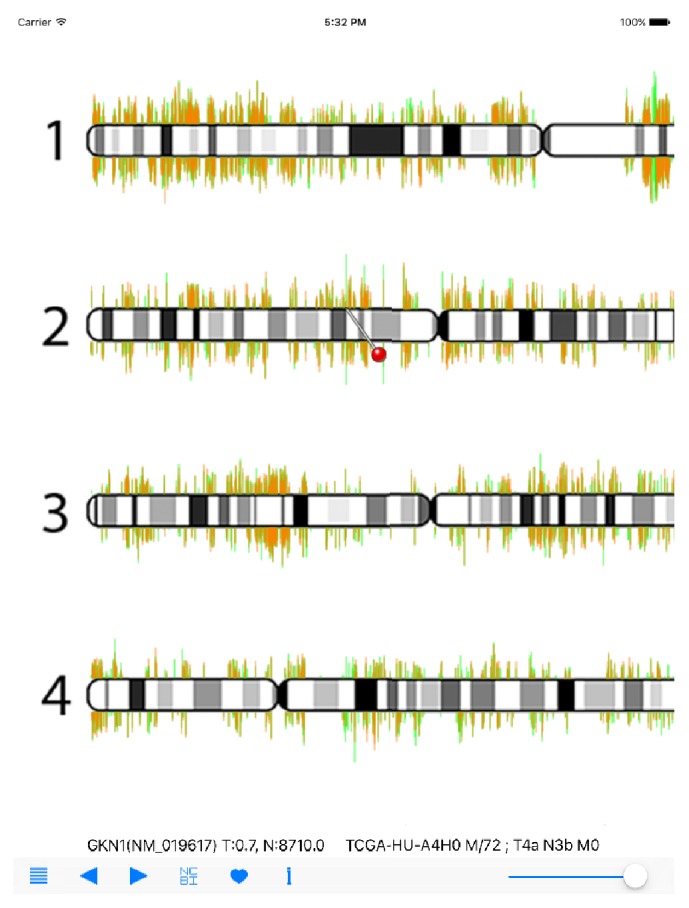
RNA-Seq Viewer display (detailed view). Users can zoom into regions of interest for detailed gene expression comparison. A red-pin marker icon will appear when users click at any gene position. The left and right navigation buttons can be used to move the red-pin icon. Gene name (symbol) and RPKM values are also displayed for interrogation. The GKN1 gene is displayed here.

The navigational and functional buttons are arranged on the bottom panel. Users can choose specific samples of a given cancer type by using the list icon on the left. Once selected, the cancer gene expression information of an individual patient is loaded and displayed. The TCGA sample ID, sex/age, and cancer stage information is also displayed (*TCGA-HU-A4H0 M/72; T4a N3b M0* in Figure 1). The gene expression value of cancer tissue is illustrated by a light-red line, and the expression value of normal tissue is illustrated by the overlapping light-green line in the background. By adjusting the intensity bar on the right of navigation panel, users can smoothly dim the light-red lines, which allows users to better compare the expression difference, especially on the overexpressed genes in cancer tissues. Users then can zoom into the regions of interest with multitouch gestures. Once a region of interest is identified, users can point-click on the gene(s) of interest to activate a red-pin icon and detailed gene symbol and RefSeq ID information as well as the expression values in tumor and normal tissues were presented (*GKN1(NM_019617) T:0.7, N:8710.0* in Figure 2). The RPKM values of tumor (T) and normal (N) tissues are displayed. The left and right arrow icons can then be used to move the red-pin marker to upstream or downstream adjacent genes. To learn more about the gene information, users can then click on the NCBI (National Center for Biotechnology Information) button to retrieve specified gene information online. In the navigation panel, the “i” icon will connect the users to our specific RNA-Seq Viewer webpage for user-guide information and also the processed TCGA data download page.

### 3.3. Display of miRNA-Seq Information

**Figure 3:**
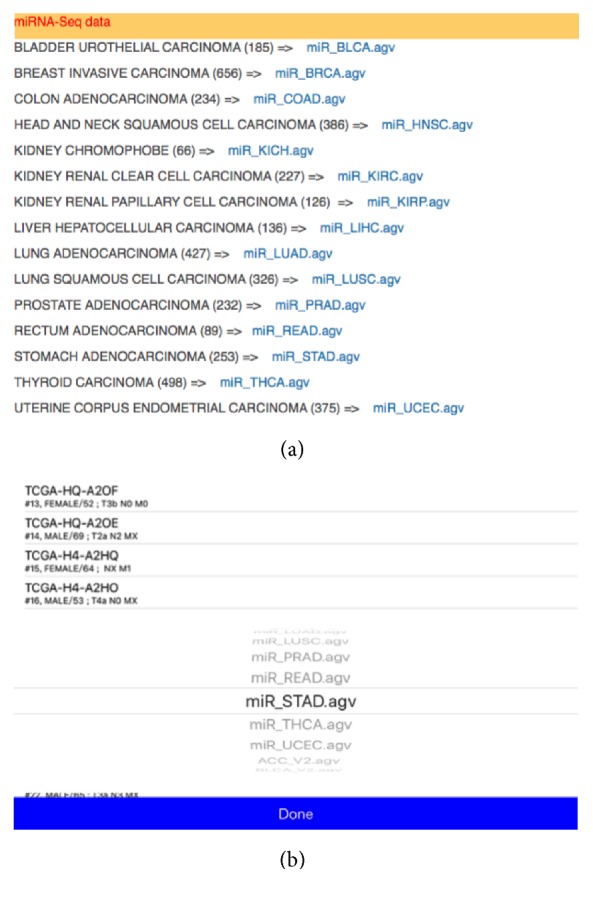
TCGA miRNA-Seq datasets. (a) Collection of miRNA-Seq samples for interrogation. The number of samples contained in each cancer type is listed in parenthesis. (b) Selection screen for different cancer types in RNA-Seq Viewer. miR_STAD (stomach cancer) is selected here.

In order to display miRNA-Seq expression information, we first need to modify the RNA-Seq Viewer to simultaneously present and display the 5p-arm and 3p-arm expression values. Because of the large numbers of genes and screen size limitation of mobile devices, the original RNA-Seq Viewer App program is designed to display sense strand genes on the top of chromosome ideogram and antisense strand genes at the bottom of chromosome. Because the numbers of human miRNAs are much lower than protein-coding genes, we decided to simply display all miRNA 5p-arm expression data on top and all 3p-arm expression data at the bottom of chromosome ideogram. So users can simply compare the expression difference between 5p-arm and 3p-arm of the same miRNA gene on the same miRNA locus position. All miRNA gene locus positions and expression values were converted to this schema and formatted into four SQLite tables. All TCGA miRNA-Seq data are freely available from our website (http://tdl.ibms.sinica.edu.tw/NGS_viewer_1/NGS_Viewer/General_Introduction.html). Because of the file size issues, this mobile App is designed to let users download only the interested cancer types from ourweb site and then synchronize data by using the iTune program. We understand that the data files transmission procedures are somewhat troublesome and awkward. This data transmission route was selected for several reasons when we designed the RNA_Seq Viewer: iTune program is the default data backup and transfer scheme for iOS devices; the NGS data files could be very large and might have issues in network transfer cost. Also, we consider the security issue if clinical data was included in the user's own data file in the future. Users are encouraged to first browse our download page and obtain the needed cancer data for interrogation. As shown in Figure 3(a), there are 15 cancer types available from our website. The sample size ranged from 66 (miR_KICH: chromophobe renal cell carcinoma) to 656 (miR_BRCA: breast carcinoma) with the file sizes around 20 to 200 MB.

**Figure 4:**
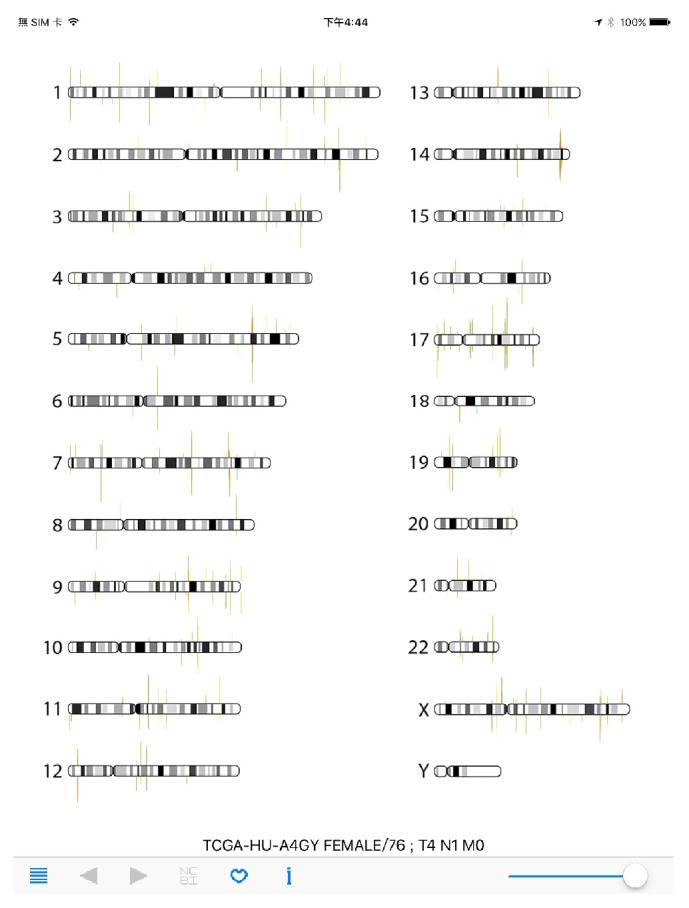
RNA-Seq Viewer display for miRNA-Seq data (overall chromosome view). A stomach cancer sample miRNA-Seq data is visualized (TCGA-HU-A4GY). The clinical information is listed on top of the navigation panel, right after the TCGA sample ID.

**Figure 5:**
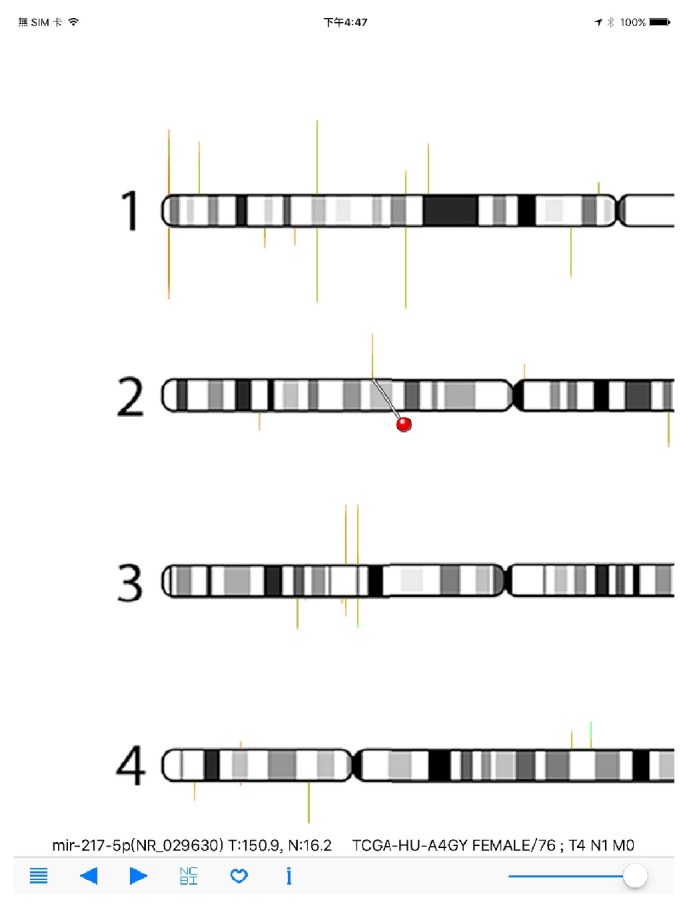
RNA-Seq Viewer display for miRNA-Seq data (detailed view). Users can zoom into regions of interest for detailed miRNA expression comparison. A red-pin icon will appear when users click at any gene position. The left and right navigation buttons can be used to move the red-pin icon. Gene name (symbol) and RPM values are also displayed for interrogation. The mir-217-5p gene is displayed here.

**Figure 6:**
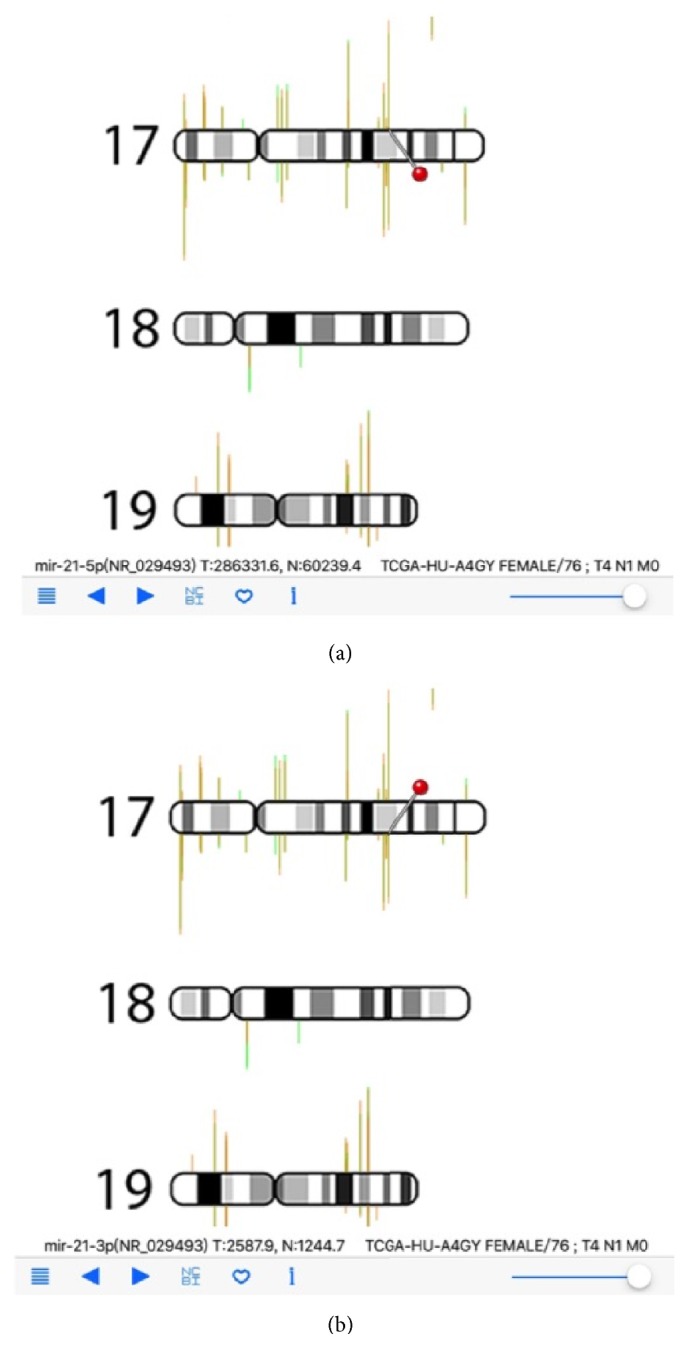
RNA-Seq Viewer display for miRNA-Seq data (detailed view). (a) miR-21-5p is selected and displayed; (b) miR-21-3p is selected and displayed.

Once cancer miRNA-Seq data files were copied to mobile devices using iTune, users can select the cancer types by using the list icon on navigation panel. Cancer type was selected first and then specific cancer sample can be chosen (Figure 3(b)). Here, as an example, the miR-STAD (stomach cancer) and sample TCGA-HU-A4G have been selected. The overall miRNA expression profile is then displayed in RNA-Seq Viewer App (Figure 4). The basic patient information is listed underneath including sex, age, and TNM stages. Comparing with protein-coding genes information (Figure 1), it is noted that the number of miRNA genes is much less and many miRNAs do form gene clusters as expected (Figures 3 and 4). Another interesting pattern observed is the preferential expression of 5p-arm or 3p-arm miRNAs (Figure 4). It is evident that many miRNAs do have dominant expression pattern on 5p-arm or 3p-arm mature miRNAs, as reported earlier [14, 19, 20, 22, 29]. For example, in Figure 5, we could observe that only the 5p-arm is expressed in miR-217 locus and this 5p-arm expression pattern is supported by the miR-217 annotation in miRBase [28]. In Figure 6, the expressions of miR-21-5p and miR-21-3p were illustrated. miR-21 is a well-known oncomiR and frequently overexpressed in tumor samples, including gastric cancer [30, 31]. In Figures 6(a) and 6(b), it is clear that both miR-21-5p and miR-21-3p are expressed in this tumor sample. The 5p-arm of miR-21 is the dominant mature miRNA here. However, it should be noticed that miR-21-3p is also upregulated in this cancer tissue. miR-21-3p was reported to have different target genes and functions [32–38]. To learn more about the miR-21-5p and miR-21-3p, one can click the NCBI icon and obtain GenBank information on this particular miRNA. To provide a convenient way to mark the cancer sample under interrogation, users can utilize the heart-shape button to label the current sample. When starting the RNA-Seq Viewer, the labeled samples will be moved to the top position of sample list, so users can easily identify the samples currently under study. Clicking on the heart-shape button again will unlabel this sample.

**Figure 7:**
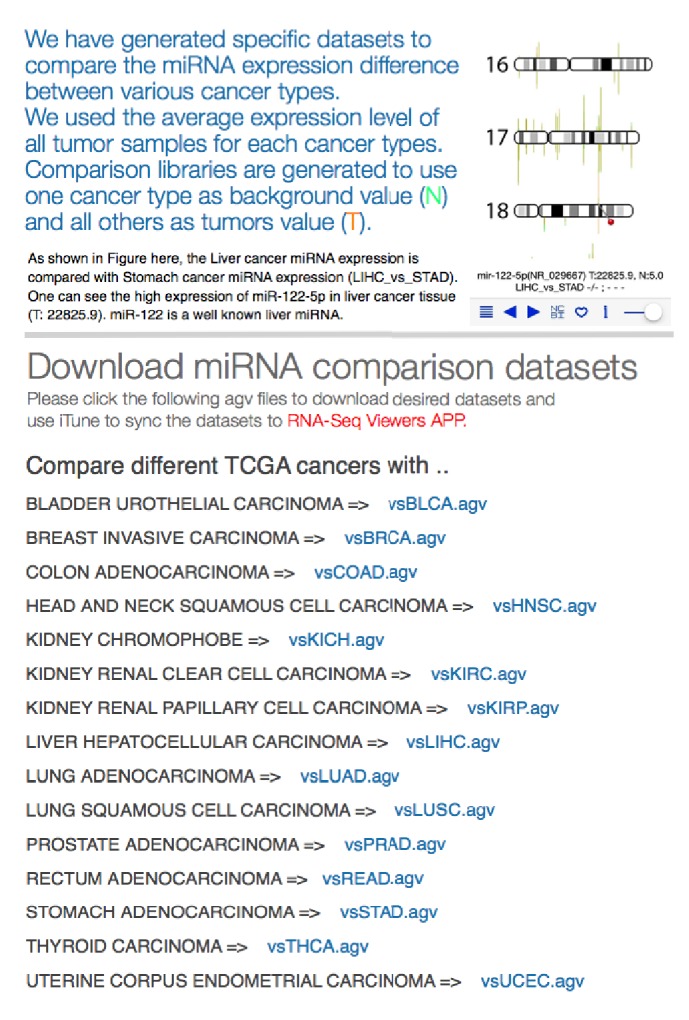
Web page for miRNA expression comparison among different TCGA cancer types. Users can obtain specific miRNA expression comparison datasets to investigate the miRNA 5p-arm and 3p-arm expression difference between different TCGA cancer types. The average expression number is calculated from all tumor samples within each cancer type and used for comparison purpose. Users can select one cancer type as background (vsCANCER.agv file) and interrogate miRNA expression against other cancer types.

In the interests of comparing the miRNA 5p-arm and 3p-arm expression difference between different cancer types, we have generated specific datasets for users to compare the miRNA expression difference between various cancer types. All miRNA expression comparison libraries can be obtained from our website (http://tdl.ibms.sinica.edu.tw/NGS_viewer_1/NGS_Viewer/miRNA_comparison.html). We used the average expression level of all tumor samples for each cancer type. Comparison libraries are generated to use one cancer type as background value (N) and all others as tumors value (T). As shown in Figure 7, the liver cancer miRNA expression is compared with stomach cancer miRNA expression (LIHC_vs_STAD). One can clearly see the higher expression of miR-122-5p in liver cancer tissue (T: 22825.9 read counts); on the contrary, miR-122-5p is lowly expressed in stomach cancer tissues (5.0 read counts). This is an anticipated result, since miR-122 is a well-known liver miRNA. As shown in Figure 7, we have generated 15 datasets for each cancer type, and users can select/download any cancer type for interrogation. (For example, vsSTAD dataset will provide comparison of miRNA expression between 14 cancer types with gastric cancer.)

In summary, our RNA-Seq Viewer does support the display of miRNA-Seq expression information and provides a user-friendly environment to interrogate the miRNA expression patterns in individual cancer sample. In addition to TCGA dataset, we also provide the SQLite table templates (user.agv) for running custom data. Users can use any SQLite tool to browse and edit their own NGS expression data table. This template uses RefSeq database information as the gene name standard and as the primary index key. The expression values can be inputted into the user.agv table template. Normal table is for the background normal tissue expression background information, and the tumor table is for each cancer sample expression level. Sample table is used to store the ID and clinical information from TCGA.

## 4. Conclusions

In conclusion, we demonstrated that large numbers of miRNA-Seq data could be visualized and interrogated efficiently on the mobile devices with our mobile App. The RNA-Seq Viewer App is useful for displaying multiple gene expression data (mRNA and miRNA) onto global chromosome context. In addition, it could be utilized as a fundamental visualization component for personalized genome information display and retrieval. In the future, under the framework of RNA-Seq Viewer, epigenetic modification information and other related gene modulation data could be integrated and visualized with this useful mobile App.
